# Distinct Neurodevelopmental Trajectories in Groups of Very Preterm Children Screening Positively for Autism Spectrum Conditions

**DOI:** 10.1007/s10803-022-05789-4

**Published:** 2022-10-23

**Authors:** Laila Hadaya, Lucy Vanes, Vyacheslav Karolis, Dana Kanel, Marguerite Leoni, Francesca Happé, A. David Edwards, Serena J. Counsell, Dafnis Batalle, Chiara Nosarti

**Affiliations:** 1 Centre for the Developing Brain, Department of Perinatal Imaging and Health, School of Biomedical Engineering and Imaging Sciences, King’s College London, London, SE1 7EH UK; 2 Department of Child and Adolescent Psychiatry, Institute of Psychiatry Psychology and Neuroscience, King’s College London, 16 De Crespigny Park, London, SE5 8AF UK; 3 Wellcome Centre for Integrative Neuroimaging, FMRIB, Nuffield Department of Clinical Neurosciences, University of Oxford, Oxford, OX3 9DU UK; 4 Social, Genetic and Developmental Psychiatry Centre, Institute of Psychiatry Psychology and Neuroscience, King’s College London, London, SE5 8AF UK; 5 Department of Forensic and Neurodevelopmental Sciences, Institute of Psychiatry Psychology and Neuroscience, King’s College London, London, SE5 8AF UK

**Keywords:** Autism spectrum conditions, Developmental delay, Very preterm birth, Structural MRI

## Abstract

Very preterm (VPT; < 33 weeks’ gestation) toddlers screening positively for autism spectrum conditions (ASC) may display heterogenous neurodevelopmental trajectories. Here we studied neonatal brain volumes and childhood ASC traits evaluated with the Social Responsiveness Scale (SRS-2) in VPT-born toddlers (N = 371; median age 20.17 months) sub-divided into three groups based on their Modified-Checklist for Autism in Toddlers scores. These were: those screening positively failing at least 2 critical items (*critical-positive*); failing any 3 items, but less than 2 critical items (*non-critical-positive*); and screening negatively. Critical-positive scorers had smaller neonatal cerebellar volumes compared to non-critical-positive and negative scorers. However, both positive screening groups exhibited higher childhood ASC traits compared to the negative screening group, suggesting distinct aetiological trajectories associated with ASC outcomes.

**Supplementary Information:**

The online version contains supplementary material available at 10.1007/s10803-022-05789-4.

## Introduction

The parent-rated Modified Checklist for Autism in Toddlers (M-CHAT), assessing child skills and behaviours, was developed as a screening tool for autism spectrum conditions (ASC) (Robins et al., 2001). ASC are characterised by two sets of core symptoms: (a) social communication and interaction deficits (SCI), which reflect difficulties in non-verbal social gestures, socio-emotional reciprocity and maintaining and developing social relationships, and (b) restricted interests and repetitive behaviours (RRBs), which include restricted and fixated interests, ritualised behaviours and altered sensitivity to sensory stimuli (American Psychiatric Association, 2013). According to the original M-CHAT scoring criteria, a positive M-CHAT screening is obtained when a child fails two or more ‘critical’ items within a set of six (e.g., “Does your child imitate you?”, “Does your child take an interest in other children?”), or three or more items overall (Robins et al., 2001). However, research in low-risk toddlers has more recently led to the recommendation of abandoning these criteria in favour of a total number of items failed, as this approach has been shown to improve the tool’s sensitivity to identify a later ASC diagnosis (Chlebowski et al., 2013).

Studies in high-risk samples using the original screening criteria have shown that very preterm (VPT; < 32 weeks’ gestation) and extremely preterm (EPT; < 28 weeks’ gestation) born toddlers are more likely to screen positively on the M-CHAT (21–25%; Limperopoulos et al., 2008; Kuban et al., 2009), compared to full-term born toddlers (5.7%; Kleinman et al., 2008). These findings, together with those showing a higher prevalence of ASC diagnoses in children born VPT (7%) compared to those born at term (1.5%; Joseph et al., 2017; Agrawal et al., 2018), suggest that VPT children may be vulnerable to experiencing both subthreshold and clinical core ASC symptoms. However, in high-risk EPT/VPT toddlers the interpretability of the M-CHAT screening has been questioned (Luyster et al., 2011; Moore et al., 2012), as these children tend to display impaired social and communication skills, which are shared features of both the so-called “preterm behavioural phenotype” (Johnson & Marlow, 2011) and ASC traits (American Psychiatric Association, 2013). Moore et al. (2012) suggested that the two original M-CHAT positive scoring criteria may differentiate between EPT toddlers with and without neurodevelopmental disabilities, as they found that the stricter critical positive screening criteria were associated with more severe neurodevelopmental impairments compared to the more liberal non-critical criteria (Luyster et al., 2011; Moore et al., 2012). Given the increased risk of developmental delay following preterm birth (Blencowe et al., 2013) and the frequent co-occurrence of developmental delay in ASC (Rubenstein et al., 2018), the use of the initially proposed different M-CHAT positive scoring criteria may therefore aid the identification of subgroups of EPT/VPT toddlers exhibiting distinct neurodevelopmental trajectories.

Widespread alterations in brain development associated with VPT birth (Volpe, 2009), may at least partly contribute to the increased likelihood of ASC behaviours in VPT children. Structural reductions in volume and alterations in functional connectivity in temporal, prefrontal, limbic and cerebellar regions have been observed in VPT individuals in the neonatal period and beyond (Ball et al., 2013, 2016; Fenoglio et al., 2017; Healy et al., 2013; Kanel et al., 2022; Rogers et al., 2012). Alterations in these regions have also been implicated in key components of ASC symptomatology (Alcalá-López et al., 2018; Ciarrusta et al., 2019; Gandhi & Lee, 2021; Ha et al., 2015) and in VPT neonates who develop ASC later in childhood (Eklöf et al., 2019; Padilla et al., 2017; Ure et al., 2016). However, no study to date has explored whether different M-CHAT positive scoring criteria could be used to identify subgroups of VPT toddlers who differ in terms of early brain development and ASC behaviour later in childhood.

In order to address these questions, this study had two main aims: to explore whether distinct M-CHAT screening groups (critical positive, non-critical positive and negative), which have been previously studied in relation to neurodevelopmental impairments in EPT toddlers (Moore et al., 2012), also differed in VPT toddlers in terms of (a) neonatal structural brain volumes and (b) ASC profiles later in childhood. Exploratory analyses were further conducted to probe the role of developmental delay in shaping the childhood trajectory for ASC traits in the different screening groups, with the use of mediation and moderation analyses.

Our first hypothesis was that both M-CHAT positive screening groups (i.e., critical positive and non-critical positive) would display volumetric reductions at term-equivalent age in brain regions implicated in ASC symptomatology (e.g., temporal, prefrontal cortex and cerebellum) compared to the negative screening group. Our second hypothesis was that toddlers belonging to the two M-CHAT positive screening groups would display more ASC-type behaviours in childhood (age 4–7 years) than toddlers belonging to the negative screening group. Thirdly, exploratory analyses tested two competing hypotheses, namely that the critical positive scorers would either exhibit: (a) fewer ASC-like behaviours than the non-critical positive scorers, indicating that a critical positive screening may reflect developmental delay (Luyster et al., 2011; Moore et al., 2012), rather than persisting ASC behaviours, or (b) similar ASC-like behaviours to the non-critical positive scorers, indicating distinct trajectories leading to similar ASC behaviours (i.e., equifinality; Cicchetti & Rogosch, 1996).

## Methods

### Participants and Study Design

511 children born at 33 weeks’ gestational age or less (median = 30 weeks; range = 23–32 weeks), between April 2010 and July 2013, were enrolled into the “Evaluation of Preterm Imaging” study (ePrime; EudraCT 2009-011602-42; Edwards et al., 2018) from 14 neonatal units across London. Inclusion criteria were: birth at or less than 33 weeks’ gestation; English-speaking parents not undergoing child protection proceedings; no magnetic resonance imaging (MRI) contraindications or major congenital malformations. Infants underwent multimodal (T1-weighted, T2-weighted, diffusion and functional) MRI at term-equivalent age (38–44 weeks) and were followed-up for behavioural and cognitive assessments at 2 (N = 484; 95% of the initial sample) and 4–7 years (N = 251; 82% of those children approached for follow-up).

Complete M-CHAT follow-up data at 2 years were available for 371 children (49.60% female; 23.18% born EPT) meeting MRI analysis inclusion criteria: i.e., postmenstrual age (PMA) at scan < 46 weeks, having no periventricular leukomalacia, parenchymal haemorrhagic infarction, or other major ischemic or haemorrhagic lesions detected on MRI or missing T2-weighted or motion corrupted images. 177 children had complete SRS-2 data at the subsequent 4–7-year follow-up (46.90% females; 25.42% born EPT). Sample characteristics are summarised in Table 1. The EPT and VPT born children within our cohort did not differ in severity of ASC traits or developmental delay (Table SM1).

**Table 1:** Sample characteristics

Variables	Median (range)	
	2-year follow-up (N = 371)	4–7-year follow-up (N = 177)
GA, weeks	30.29 (23.57–32.86)	30.29 (24–32.86)
IMD score at birth	17.71 (1.73–60.58)	16.12 (1.73–59.16)
PMA at scan	42.57 (37.86–44.86)	42.57 (38.29–44.86)
Neonatal sickness^a^	−0.30 (−1.36–2.55)	−0.35 (−1.34–2.18)
Corrected age at assessment	20.17 (18.37–29.33) months	4.59 (4.18–7.17) years

Sample characteristics (median and range) for 2-year follow-up sample with complete M-CHAT and structural MRI data and for 4–7-year follow-up sample with complete M-CHAT and SRS-2 data

*GA* gestational age, *IMD* index multiple deprivation, *PMA* postmenstrual age

^a^excluding one subject with incomplete clinical data

### MR Imaging Data

#### Data Acquisition

A 3-Tesla system (Philips Medical Systems, Best, The Netherlands) was used to acquire MR images using an 8-channel phased array head coil. A paediatrician supervised infant care during MR imaging. Pulse oximetry, temperature, and electrocardiography data were monitored throughout the session. Silicone-based putty (President Putty, Coltene Whaledent, Mahwah, NJ, USA) and neonatal earmuffs (MiniMuffs, Natus Medical Inc., San Carlos, CA, USA) were used for ear protection. Oral chloral hydrate (25–50 mg kg^−1^) was administered to infants whose parents chose sedation for the procedure (87%). High-resolution anatomical images were acquired with T2-weighted fast spin echo sequences (repetition time = 8,670 ms; echo time = 160 ms; flip angle = 90°, slice thickness = 1 mm, field of view = 220 × 220 mm^2^, voxel size = 0.86 × 0.86 × 1 mm^3^).

### Tensor Based Morphometry

Following methods described in Vanes et al. (2021) and Lautarescu et al. (2021) T2-weighted (images and tissue type segmentations) were registered to a study-specific template using ANTS software Symmetric Normalisation algorithms (Avants et al., 2011). Resultant nonlinear transformation deformation tensor fields (warps) were used to calculate deformation tensor field gradients (log-Jacobian determinant maps) as a measure of relative brain volume. Greater log-Jacobian values represent the extent of contraction voxels undergo following registration (i.e., larger volumes), while smaller values represent volume reductions (Avants & Gee, 2004). Smoothing with 4 mm full-width half-maximum Gaussian filter was applied.

### Perinatal Socio-Demographic and Clinical Data

#### Perinatal Clinical Data

With parental consent, the infant’s electronic medical records were accessed using the Standardised Electronic Neonatal Database to collect perinatal socio-demographic and clinical data. Data capturing neonatal clinical risk were collected as part of the larger ePrime study (Edwards et al., 2018), as clinical risk can exacerbate the long-term sequelae of VPT birth (Volpe, 2009). A principal component analysis (PCA) summarised 28 perinatal clinical variables explaining 72% of their variance with a single component, which was labelled ‘neonatal sickness index’, as previously described in Kanel et al. (2021), The variables with the highest factor loadings were: GA, days on total parenteral nutrition, days on continuous positive airway pressure, days on mechanical ventilation and surfactant administration. Clinical variables were coded so that increased neonatal sickness index values indicate greater clinical risk.

#### Perinatal Environmental Data

An Index of Multiple Deprivation (IMD) score was computed from the infant’s residential postcode at time of birth (Department for Communities and Local Government, 2011; https://tools.npeu.ox.ac.uk/imd/). The IMD summarises area-level information in 7 domains: income, employment, education, health, crime, housing and living environment. Higher IMD scores reflect increased deprivation in the neighbourhood, hence higher social risk.

### Behavioural and Cognitive Measures

At the 2-year follow-up, toddlers were assessed with the parent-rated M-CHAT. Critical positive M-CHAT screening was defined by failing any 2 out of the 6 critical items: “Does your child take an interest in other children?”, “Does your child ever use his/her index finger to point, to indicate interest in something?”, “Does your child ever bring objects over to you to show you something?”, “Does your child imitate you?”, “Does your child respond to his/her name when you call?”, “If you point at a toy across the room, does your child look at it?” (Robins et al., 2001). The definition used by Moore and colleagues (Moore et al., 2012) was used to define ‘non-critical’ positive screening: failing any 3 or more items, but fewer than two critical items. Toddlers not meeting either of these criteria received a negative M-CHAT screening.

The following measures were used to assess infants’ development at 2 years: the Bayley Scales of Infant Development, Third Edition (Bayley-III; Bayley, 2006), which evaluates expressive and receptive language, fine and gross motor skills and composite cognitive scores, and the Parent Report of Children's Abilities Revised (PARCA-R; Johnson et al., 2004; Saudino et al., 1998), which evaluates toddlers’ vocabulary and sentence complexity and non-verbal cognitive skills.

To reduce the dimensionality of the behavioural outcome data, a PCA was performed. All Bayley-III and PARCA-R index scores were included in the model and the elbow-method was used to determine the number of principal components explaining most of the variance in the data. A scree plot showing the percentage of variance explained by each principal component (i.e., eigenvalues) suggests an optimal number of 2 principal components (Supplementary Information eFig. SM1), jointly explaining a cumulative 69% of total variance. Pearson correlations between each of the two resultant principal components and individual index scores were used to define each of the components. PC1 correlated negatively with all Bayley-III and PARCA-R items, resulting in a component summarising global (cognitive, language and motor) developmental delay, while PC2 correlated positively with language items (PARCA-R sentence complexity and vocabulary scores and Bayley-III expressive language scores) and showed negative correlations with gross and fine motor Bayley-III scores (Supplementary Information eFig. SM2). The first principal component was labelled as a global ‘developmental delay’ index and the second as a ‘language’ index.

At the 4- to 7-year-old follow-up, the Social Responsiveness Scale, Second Edition (SRS-2; Constantino & Gruber, 2012) was administered to measure core ASC symptoms in early childhood; it contains a Social Communication/Interaction (SCI) and a Restricted/Repetitive Behaviour (RRB) subscale. The SCI subscale indexes deficits in behaviours relating to social awareness, cognition, communication, and motivation, and the RRB subscale reflects the severity of restrictive and repetitive patterns of behaviours and interests (Constantino & Gruber, 2012). The SRS-2 shows good internal consistency (Cronbach’s alpha = 0.92 and 0.93 for females and males, respectively) as well as construct, convergent and concurrent validity in 5–8-year-old children from the United Kingdom (Wigham et al., 2012).

### Statistical Analyses

#### Univariate Phenotypic Group Differences

Statistical analyses were conducted using R (version 3.6.1). Non-parametric Kruskal–Wallis tests compared continuous measures (developmental profiles at 2 years, socio-demographic and clinical profiles at birth and SRS-2 SCI and RRB scores at 4–7 years) between M-CHAT groups (*onewaytests* R package; Dag et al., 2018). For categorical variables (sex), Chi-squared test was used. Post-hoc pairwise comparisons were made for variables showing a significant effect of group (p < 0.05). Post-hoc pairwise between-group median differences (for continuous variables) or odds ratios (for categorical variables) were reported and post-hoc pairwise comparison p-values were corrected using False Discovery Rate (Benjamini & Hochberg, 1995). A generalised linear model with 10,000 permutations investigating the effect of M-CHAT group on SCI and RRB scores and correcting for covarying effects of developmental delay, sex, IMD and neonatal sickness index, was also tested (p-permute; https://github.com/lucasfr/grouped_perm_glm).

#### Childhood Symptoms Exceeding Clinical Cut-Offs For Autism

Having a total SRS-2 T-score greater than or equal to 76 is considered to be clinically meaningful as it indicates a high likelihood of receiving an ASC diagnosis (Constantino & Gruber, 2012). We calculated the number of children scoring above the SRS-2 clinical cut-off within each M-CHAT group. Sample size calculations were then performed in order to ascertain whether the sample size was adequate for predictive validity analyses (Linden, 2020). The following measures were used as inputs in the sample size calculation: expected sensitivity/specificity (52%/84% respectively; Kim et al., 2016), prevalence in current sample (2%) and confidence interval for estimates (95%-CI with CI-width = 0.1).

#### Mass-Univariate Group Differences in Brain Volume

Differences in voxel-wise volume (log-Jacobian) measures at term-equivalent age between the three M-CHAT screening groups were investigated using general linear models correcting for sex, PMA, IMD and neonatal sickness index. FMRIB Software Library (FSL)’s *randomise* function with 10,000 permutations per run was used for non-parametric permutation testing with Threshold-Free Cluster Enhancement and controlled for family-wise error rate. Significance was set at p < 0.05 per contrast, given the exploratory nature of the analysis.

Post-hoc analyses investigating associations between neonatal brain volumes showing between-group differences and ASC traits in childhood are described in the supplemental information (Table SM2). We also explored associations between M-CHAT total items failed and neonatal whole-brain Jacobian values.

#### Testing the Role of Developmental Delay

To test for a potential role of early developmental delay in explaining (mediating) or exacerbating (moderating) later group differences in core ASC symptoms, analyses using general linear models were conducted.

Specifically, where between-group differences in later ASC symptoms (SRS-2 SCI or RRB) at 4–7 years were observed, we tested whether these differences were significantly mediated by developmental delay at 2 years. In addition, to test whether developmental delay at 2 years shows a differential relationship with later ASC symptoms in the separate M-CHAT groups, we tested for effects of developmental delay and M-CHAT screening, as well as their interaction, on SRS-2 SCI and RRB scores. Both mediation and moderation analyses used sex, IMD, and neonatal sickness index as confounders. Mediation was tested via bootstrapping of the indirect effect (based on 5000 bootstrap samples) using the R ‘mediation’ package (Tingley et al., 2014). To adjust for multiple comparisons due to two separate outcome variables (SRS-2 RRB and SCI), 97.5%-confidence intervals (97.5%-CIs) were generated. P-values with a corrected significance threshold of p < 0.05/2 (i.e., 0.025) were estimated from non-parametric permutation testing with 10,000 permutations (p-permute; https://github.com/lucasfr/grouped_perm_glm).

## Results

### Comparing M-CHAT Groups on Socio-Demographic, Clinical and Developmental Outcomes

Median scores and F-statistics and p-values comparing M-CHAT group socio-demographic and clinical outcomes are summarised in Table 2 and developmental profiles and Bayley-III and PARCA-R composite scores in Table 3. The three groups did not differ in corrected age at M-CHAT assessment, PMA at scan, GA at birth, birthweight, neonatal sickness index or language development (Tables 2; 3).

**Table 2:** Socio-demographic and clinical profiles for M-CHAT groups

Variable	Median (Interquartile range)			F-statistic; p-value
	Negative (N = 267; 143 female)	Non-critical positive (N = 77; 33 female)	Critical positive (N = 27; 8 female)	
Socio-demographic variables				
Corrected age at 2 years, months median (IQR)	20.20 (0.67)	20.13 (0.70)	20.03 (0.37)	F = 2.11; p = 0.310
Corrected age at 4–7 years, years	4.59 (0.58)	4.67 (0.90)	4.59 (0.91)	F = 4.78; p = 0.092
PMA at scan, weeks	42.57 (2.00)	42.71 (2.14)	42.57 (1.50)	F = 5.27; p = 0.072
IMD score at birth	16.54 (17.00)	19.92 (15.71)	25.87 (15.79)	F = 7.63; p = 0.022*
GA, weeks	30.29 (3.50)	30.86 (4.14)	28.86 (3.36)	F = 3.58; p = 0.167
Birthweight, grams	1315 (570.00)	1270 (650.00)	1040 (485.00)	F = 3.27; p = 0.196
Neonatal sickness index^a^	−0.36 (1.71)	−0.45 (1.49)	0.55 (1.59)	F = 3.91; p = 0.142

*GA* gestational age at birth, *IMD* index multiple deprivation, *PMA* postmenstrual age

*p < 0.05

^a^excluding one subject with incomplete clinical data

**Table 3:** Developmental profiles and Bayley-II and PARCA-R composite scores for M-CHAT groups

Variable	Median (Interquartile range)			Statistic; p-value
	Negative (N = 267)	Non-critical positive (N = 77)	Critical positive (N = 27)	
Developmental profiles at 2 years^b^				
Developmental delay	−0.57 (2.67)	0.88 (2.25)	2.86 (2.76)	F = 57.40; p < 0.001***
Language	−0.02 (1.28)	−0.09 (1.28)	0.28 (1.54)	F = 2.33; p = 0.313
Bayley-III composite scores at 2 years^b^				
Cognitive	95.00 (18.75)	90.00 (20.00)	82.50 (20.00)	F = 27.28, p < 0.001***
Language	97.00 (20.00)	83.00 (20.00)	69.50 (14.00)	F = 45.36, p < 0.001***
Motor	100.00 (9.00)	94.00 (12.00)	82.00 (18.00)	F = 45.14, p < 0.001***
PARCA-R composite scores at 2 years^b^				
Vocabulary	19.00 (18.05)	10.00 (11.25)	3.00 (5.75)	F = 34.52; p < 0.001***
Sentence complexity	5.00 (6.00)	3.00 (4.25)	4.00 (2.00)	F = 36.21; p < 0.001***

*IMD* index multiple deprivation, *PARCA-R* parent report of children’s abilities-revised

***p < 0.001

^b^excluding 31 subjects with incomplete developmental data at 2-years

Variables showing a significant group effect were investigated for pairwise group differences and median difference and post-hoc p-values for between-group differences are reported in Table 4. In summary, social risk (IMD scores) was lower in negative M-CHAT scorers than critical positive scorers, but did not differ between other groups. Of the three groups, negative M-CHAT scorers had the lowest developmental delay scores (indicating better language, cognitive and motor scores), the critical positive scorers showed the greatest developmental delay and non-critical positive scorers showed intermediate developmental delay scores. There was an overall difference in male-to-female ratios between the different M-CHAT sub-groups (Chi-squared = 7.38; p = 0.025), although all pairwise comparisons were not statistically significant (p > 0.05; M-CHAT negative group compared to non-critical and critical groups; odds ratio = 1.54 and 2.74, p = 0.147 and 0.053, respectively; non-critical group compared to the critical group; odds ratio = 1.78, p = 0.226). The proportion of females in the M-CHAT negative, non-critical positive and critical positive groups were 53.56%, 42.86% and 29.63% respectively.

**Table 4:** Post-hoc pairwise differences (between the three M-CHAT screening groups) for variables with a significant effect of M-CHAT group

Variable	Median difference (p-value)		
	Negative vs non-critical positive	Negative vs critical positive	Non-critical positive vs critical positive
IMD score at birth	−3.38 (p = 0.646)	−9.33 (p = 0.020)*	−5.95 (p = 0.039)*
Developmental delay	5.00 (p = 0.003)**	−3.43 (p < 0.001)***	−1.98 (p < 0.001)***
Bayley-III: Cognitive	14.00 (p < 0.001)***	12.50 (p < 0.001)***	7.50 (p = 0.006)**
Bayley-III: Language	6.00 (p < 0.001)***	27.50 (p < 0.001)***	13.50 (p < 0.001)***
Bayley-III: Motor	9.00 (p = 0.001)***	18.00 (p < 0.001)***	12.00 (p < 0.001)***
PARCA-R: Vocabulary	2.00 (p < 0.001)***	16.00 (p < 0.001)***	7.00 (p = 0.003)**
PARCA-R: Sentence complexity	−1.45 (p < 0.001)***	4.00 (p < 0.001)***	2.00 (p = 0.010)**

Between-group statistics (median differences for variables with significant effects of M-CHAT group) and pairwise comparison p-values are reported for variables showing significant effects of M-CHAT group

*IMD* index multiple deprivation, *PARCA-R* parent report of children’s abilities-revised

*p < 0.05; **p < 0.010; ***p < 0.001

### Differences In Brain Volume at Term-Equivalent Age Between M-CHAT Groups

**Fig. 1:**
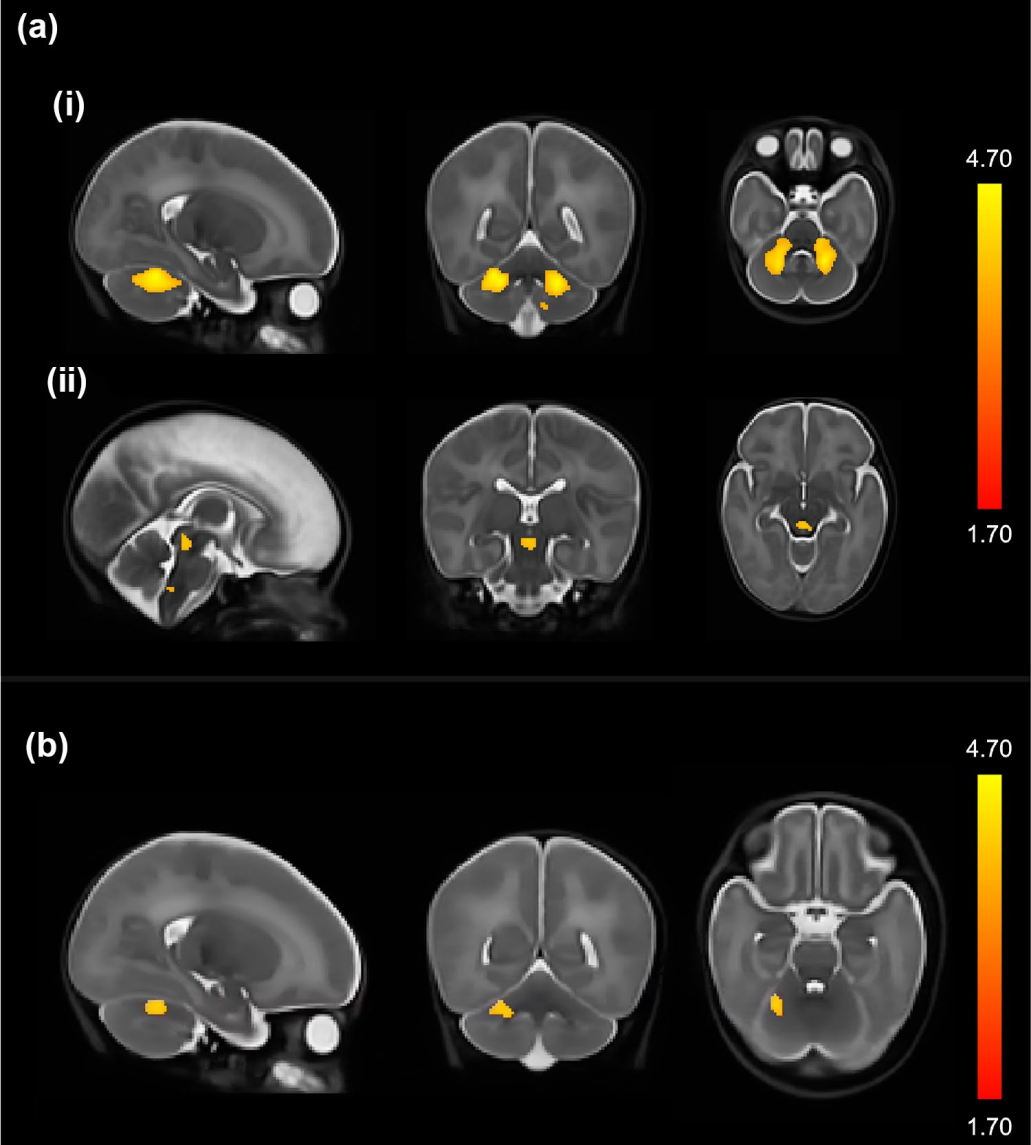
Study-specific brain template overlaid with coloured T-statistics map of brain regions significantly smaller in the M-CHAT critical positive group compared to **a** the M-CHAT negative group and **b** the M-CHAT non-critical positive group

Voxel-wise group comparisons of relative brain volume (correcting for sex, PMA, IMD and neonatal sickness index) showed that critical positive scorers had reduced regional volume in the bilateral deep cerebellar nuclei, middle cerebellar peduncles and midbrain and medulla regions of the brainstem compared to negative scorers (Fig. 1A). Critical positive scorers also showed volume reductions in an overlapping region in the right cerebellar nuclei compared to the non-critical positive group (Fig. 1B). Coloured T-statistic maps of regions showing significant differences between critical and negative scorers are depicted in Fig. 1A and between critical and non-critical scorers in Fig. 1B, where T-statistic values ranging from 1.70 to 4.70 are denoted by the colour bar. Non-parametric permutation tests with Threshold-Free Cluster Enhancement controlling family-wise error rate were used to identify between-group differences (p < 0.05).

There were no significant associations between regional cerebellar volumes and ASC traits at 4–7 years of age in any of the three groups (Table SM2). Furthermore, when investigating the association between M-CHAT total items failed and neonatal whole-brain Jacobians values, we found no significant correlations (p > 0.05).

### ASC Traits in Childhood

**Fig. 2:**
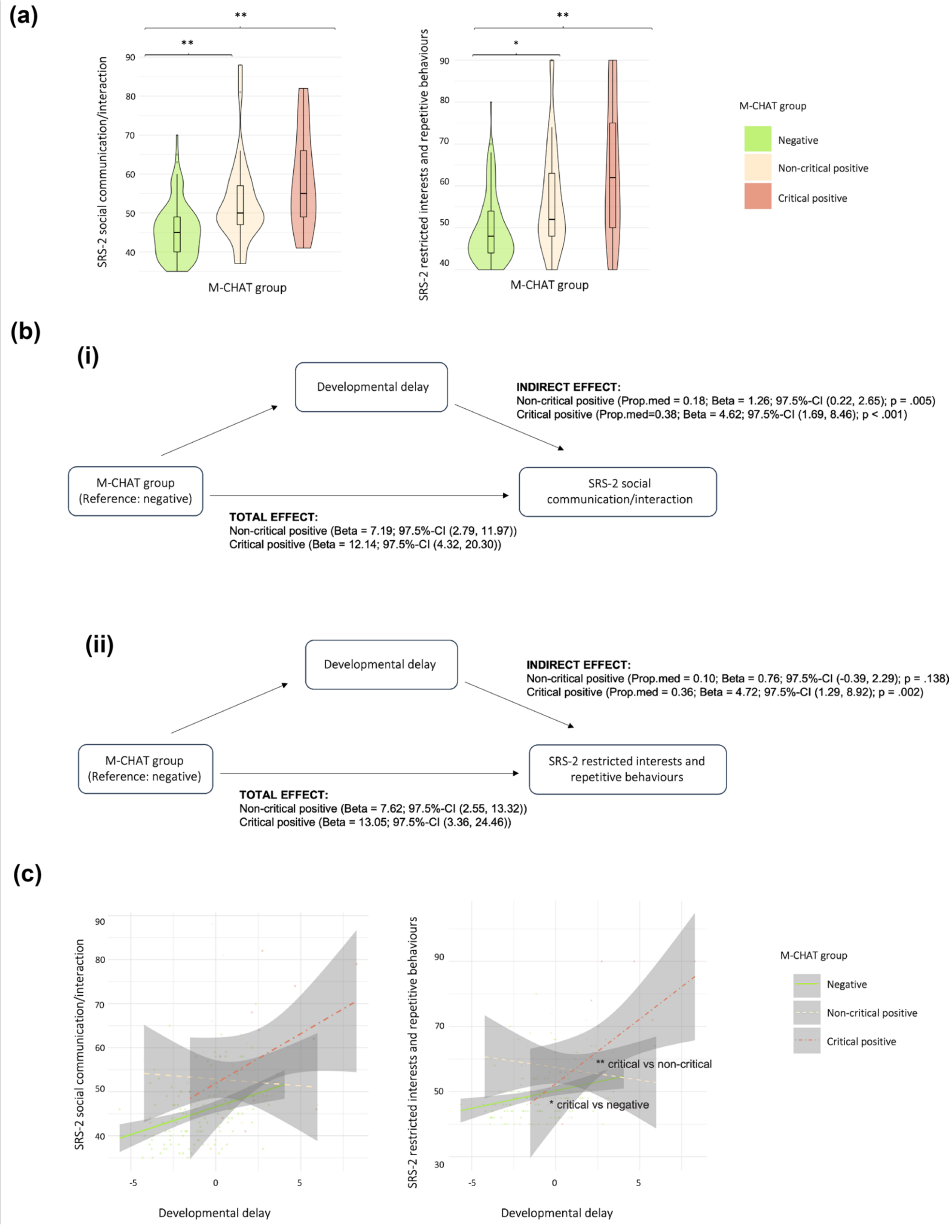
**a** SRS-2 SCI/RRB median differences between M-CHAT screening groups, **b** the mediating effect of developmental delay on the relationship between M-CHAT and SCI/RRB and **c** the moderating effect of the M-CHAT group × developmental delay interaction on SCI/RRB. *p <0.025; **p < 0.010

A significant effect of group on SRS-2 SCI and RRB was observed (Table 5). Pairwise comparisons showed that both M-CHAT (critical and non-critical) positive groups had higher SCI and RRB scores compared to the negative group; however, SCI and RRB scores did not differ between the two positive groups (Table 6; Fig. 2A). These findings did not change after adjusting for sex, IMD, neonatal sickness index and developmental delay.

**Table 5:** ASC traits at 4–7 years in the M-CHAT screening groups

Variable	Median (Interquartile range)			F-statistic; p-value
	Negative (N = 130)	Non-critical positive (N = 32)	Critical positive (N = 15)	
SRS-2 SCI	45.00 (9.50)	49.50 (10.00)	55.00 (17.00)	F = 17.69; p < 0.001***
SRS-2 RRB	4.00 (5.00)	5.50 (7.25)	11.00 (12.50)	F = 14.02; p < 0.001***

*RRB* restricted interests and repetitive behaviours, *SCI* social communication/interaction, *SRS-2* social responsiveness scale, second edition

***p < 0.001

**Table 6:** Post-hoc pairwise differences (between the three M-CHAT screening groups) for ASC traits with a significant effect of M-CHAT group

Variable	Median difference (p-value)		
	Negative vs non-critical	Negative vs critical	Non-critical vs critical
SRS-2 SCI	−4.50 (p = 0.006)**	−10.00 (p = 0.001)***	−5.50 (p = 0.133)
SRS-2 RRB	−1.50 (p = 0.020)*	−7.00 (p = 0.005)**	−5.50 (p = 0.122)

Between-group statistics (median differences for variables with significant effects of M-CHAT group) and pairwise comparison p-values are reported for SRS-2 ASC trait outcomes showing significant effects of M-CHAT group

*RRB* restricted interests and repetitive behaviours, *SCI* social communication/interaction, *SRS-2* social responsiveness scale, second edition

*p < 0.05; **p < 0.01; ***p < 0.001

5 children out 177 (2.8%) had SRS-2 scores exceeding clinical cut-offs for autism (i.e., having SRS-2 total T-scores greater than or equal to 76), where 2 belonged to the non-critical positive group and 3 belonged to the critical positive group. Formal predictive validity analyses were not performed, as sample size analyses estimated a larger sample (N = 480) would be needed to carry them out.

### Mediating and Moderating Effects of Developmental Delay on ASC Traits

#### Mediation Analyses

Due to the significant differences observed in both SRS-2 SCI and RRB childhood scores between negative scorers and the two positive groups, we tested whether pairwise group differences were at least partially accounted for by developmental delay. Developmental delay significantly partially mediated differences in SCI when comparing negative to critical (indirect effect 97.5%-CI = 1.69, 8.46; p < 0.001) and non-critical positive groups (indirect effect 97.5%-CI = 0.22, 2.65; p = 0.005; Fig. 2Bi). Proportion mediated (Prop.med) was 0.18 for M-CHAT negative vs non-critical positive group, and 0.38 for M-CHAT negative vs critical positive group.

Developmental delay also significantly partially mediated group differences in RRB when comparing the negative to the critical positive (indirect effect 97.5%-CI = 1.29, 8.92; p = 0.002; Prop.med = 0.36), but not to the non-critical positive group (indirect effect 97.5%-CI = -0.39, 2.29; p = 0.138; Prop.med = 0.18; Fig. 2Bii). Mediation analyses for the two positive groups were not conducted, as these did not differ significantly in SCI or RRB scores.

#### Moderation Analyses

A linear model regressing SCI scores on M-CHAT grouping, developmental delay, and their interaction (M-CHAT × developmental delay), controlling for sex, IMD and neonatal sickness index, found no significant interaction, *F*(2, 159) = 2.73, *p* = 0.069; p-permute = 0.074, indicating that the effect of developmental delay on SCI scores was similar in the three M-CHAT groups.

In contrast, a model regressing RRB scores on M-CHAT grouping, developmental delay, and their interaction (M-CHAT × developmental delay), controlling for sex, IMD, and neonatal sickness index, revealed a significant overall interaction, *F*(2,159) = 6.73, *p* = 0.002; p-permute = 0.003. Re-coding each group as the reference category showed this was due to a significant interaction when comparing the critical positive group to both negative and non-critical positive groups (Table 7). The M-CHAT critical positive group had a stronger (positive) association between developmental delay and RRB scores compared to both negative and non-critical positive groups (Fig. 2C).

**Table 7:** M-CHAT x developmental delay interaction on SRS-2 RRB scores

Interaction term	Beta	SE	T-statistic	97.5%-CI	Permutation p-value
M-CHAT (non-critical positive vs negative) × developmental delay	−2.00	1.00	−2.01	(−4.25, 0.26)	0.047
M-CHAT (critical positive vs negative) × developmental delay	2.95	1.11	2.66	(0.44, 5.46)	0.013*
M-CHAT (critical positive vs non-critical positive) × developmental delay	4.95	1.35	3.66	(1.89, 8.00)	0.001***

Table summarising, beta, standard error (SE), T-statistic, 97.5% confidence intervals (97.5%-CI) and non-parametric permutation testing p-values for effect of interaction terms between M-CHAT group and developmental delay on RRB scores

*RRB* restricted interests and repetitive behaviours, *SE* standard error, *SRS-2* social responsiveness scale, second edition

*p < 0.025; ***p < 0.001

## Discussion

This study investigated neonatal brain volumes and ASC traits in childhood in VPT children sub-divided into three groups, based on their M-CHAT screening outcomes (negative, non-critical positive and critical positive). Addressing our first aim, we found that the three groups exhibited differences in structural brain volumes at term-equivalent age, indicating distinct early biological phenotypes. The critical positive scorers displayed smaller volumes in cerebellar and brainstem regions compared to negative scorers, and smaller regional cerebellar volumes compared to non-critical positive scorers. Addressing our second aim, we found that while both positive groups showed higher ASC core symptom scores (RRB and SCI) relative to negative scorers, there were no significant differences between the two positive groups. However, the critical positive scorers showed greater developmental delay compared to the other two groups. Taken together our findings suggest that the two M-CHAT positive groups do not differ in the severity of childhood ASC traits and we speculate that they may be following distinct aetiological trajectories leading to similar ASC traits in childhood (i.e., equifinality; Cicchetti & Rogosch, 1996).

The early differences in regional brain volumes found between the positive M-CHAT groups, provide evidence for potentially distinct biological mechanisms underlying later ASC outcomes in a subset of VPT children. The critical positive M-CHAT group showed reduced relative volumes within regions of the right cerebellar nuclei compared to the non-critical positive group, and more widespread reductions in bilateral cerebellar nuclei and brainstem (medulla oblongata and midbrain) volumes compared to the negative group. The cerebellum is known to play a critical role in coordinating motor, sensory and cognitive abilities, which are also impacted in ASC (Wang et al., 2014). Cerebellar alterations have been associated with ASC symptomatology/traits both in animal and human studies. Cellular cerebellar pathology has been linked to increased ASC-like behaviours in mice (Tsai et al., 2012), smaller white matter volume in the cerebellum has been described in adults with ASC (Toal et al., 2010) and number and density of Purkinje cells has been shown *post-mortem* to be altered in individuals with ASC (Wegiel et al., 2010, 2014). In VPT samples, cerebellar volume reductions in childhood (Ure et al., 2016) and increased cerebellar haemorrhagic injury in infancy (Limperopoulos et al., 2007) were displayed in those with an ASC diagnosis or those screening positively on the M-CHAT. In both studies, VPT children with ASC diagnoses (Ure et al., 2016) and with cerebellar injury (Limperopoulos et al., 2007) had a high prevalence of developmental delay. Similar to the results of the aforementioned studies, which show cerebellar volume reductions in groups of children with increased developmental delay, we also found that the group exhibiting the most severe developmental delay (i.e., M-CHAT critical positive group) had smaller cerebellar volumes relative to the non-critical positive and negative groups.

The brainstem, which in this study showed reduced regional volumes in the M-CHAT critical positive relative to the M-CHAT negative group, is an early phylogenetic region of the brain known to be important for primitive functions such as arousal, respiration, and physiological regulation, although there is some evidence of its role in self-regulatory behaviours (Geva & Feldman, 2008; Geva et al., 2014). Of particular relevance to the current findings, Geva et al. (2013) showed that brainstem functioning in VPT infants was associated with social integration abilities assessed using modulation of gaze in response to social stimuli at 4 months. Furthermore, white matter reductions in the brainstem have been observed in adults with ASC compared to controls (Toal et al., 2010) and early histological work investigating brainstem injury, specifically in the motor cranial nerve nuclei, suggest that early alterations to this brain region may contribute to the onset of autism later in life (Rodier, 2002; Rodier et al., 1996, 1997). The cerebellar nuclei and brainstem (medulla oblongata and midbrain) interact with one another to facilitate sensory, motor and regulatory processes (Watson et al., 2013). The olivary complex in the medulla sends fibres to the cerebellar nuclei allowing for integration of motor and sensory information and has been found to be altered *post-mortem* in individuals with ASC (Wegiel et al., 2013). Interactions between the midbrain and the olivary-cerebellar complex have been discussed in the context of processes relating to “survival networks”, which involve behavioural (social, motor and sensory) regulation in response to emotional and environmental stimuli (Watson et al., 2013), which are core processes in ASC symptomatology. In light of these findings, we tentatively speculate that the regional brain alterations we observed in the M-CHAT critical positive compared to the negative group may represent a biological mechanism contributing to the increased RRB and SCI behaviours seen in this group.

Findings showing neonatal regional brain volume reductions as well as increased developmental delay observed in critical compared to non-critical positive scorers, despite the two groups showing similar childhood ASC traits (SCI/RRB), probed us to further investigate developmental delay in relation to ASC traits in the different groups. Results showed that developmental delay had both an explanatory (i.e., mediating) effect, as well as an exacerbating role (i.e., moderating effect) specific to RRB scores, in the critical positive group (but not SCI scores). These results suggest that VPT toddlers meeting the critical positive M-CHAT criteria may, therefore, represent an aetiologically distinct subgroup of children whose developmental difficulties increase their likelihood of developing RRB symptoms. Differences in RRB traits between preterm and term-born children have been previously explained by differences in IQ (Johnson et al., 2010), further supporting the notion that developmental delay may contribute to elevated childhood RRB traits. However, it is worth noting that in our study RRB traits were only partially explained by developmental delay, as the higher childhood RRB scores in M-CHAT critical positive scorers compared to negative and non-critical positive scorers were significant after correcting for developmental delay.

The two M-CHAT positive screening groups did not differ in SCI scores, but had elevated SCI scores relative to the negative screening group, which were significant even after correcting for developmental delay. This indicates that developmental delay at least partially contributes to the SCI difficulties seen in both M-CHAT positive groups, which is in line with observations in children with ASC (Hirosawa et al., 2020). However, developmental delay in the current study did not moderate the relationship between M-CHAT group and SCI difficulties, suggesting that the effect of developmental delay on subsequent SCI outcomes was similar in all three groups. These results motivate future studies to investigate which additional biological and/or environmental factors could be driving similar SCI outcomes in the two positive groups, who showed distinct neurodevelopmental profiles early in life.

This study’s findings tentatively suggest that the M-CHAT in VPT toddlers represents a useful tool to identify individuals with an increased likelihood of displaying ASC traits in childhood. This is firstly supported by findings showing increased developmental difficulties in both M-CHAT positive groups compared to the negative group, as well as higher median RRB and SCI scores, even after accounting for developmental delay. Secondly, as all children scoring above SRS-2 clinical cut-off thresholds (N = 5, or 2.8% of the sample) belonged to both M-CHAT positive groups, this study suggests that the tool has high sensitivity in VPT cohorts. Finally, although most positive scorers did not exceed the SRS-2 clinical cut-off score for ASC, they did exhibit subthreshold socio-emotional difficulties which are reportedly common amongst VPT children (Johnson & Marlow, 2011).

This study has several limitations, the main being that ASC diagnoses were not systematically evaluated at childhood assessment (4–7 years), although a current follow-up study is now collecting these data at 8–9 years. Moreover, sample size analyses showed we did not have an adequate number of participants to perform formal predictive validity analyses, as the number of children in our sample exceeding SRS-2 clinical cut-off scores were very few. Another limitation of this study is that the results presented are not generalisable to children with major brain lesions, who are likely to have more severe developmental impairments later in life (Volpe, 2009), but were not included in the current analyses. Future studies could therefore focus on better understanding the relationship between developmental delay following major brain injury and later ASC behaviours/traits. In addition, other neuroimaging modalities measuring brain functional and structural connectivity were not investigated, and future studies could use a multi-modal approach to provide greater insight into the biological underpinnings associated with the distinct pathways to increased likelihood of developing ASC following VPT birth. Furthermore, while in this paper we consider separate M-CHAT groups, it is plausible that the three groups may lie on a continuum. The non-critical positive scorers' developmental outcomes were in fact intermediate between the two other groups, with the negative scorers showing the best outcomes and the critical positive scorers showing the poorest outcomes.

In summary, our results highlight the distinct early developmental and neurobiological characteristics in M-CHAT critical versus non-critical positive scorers, despite them presenting with similar childhood ASC-symptom profiles. Our results also further highlight the importance of interpreting M-CHAT screenings in combination with other developmental measures when assessing VPT toddlers. Identifying biomarkers and developmental trajectories of later ASC outcomes could guide clinicians and researchers to devise personalised interventions aimed at supporting children’s development based on their distinct phenotypic presentations preceding the onset of ASC symptoms.

### Supplementary Information

Below is the link to the electronic supplementary material.

Supplementary file1 (DOCX 225 KB)

## Declarations

### Data Availability

Access to the dataset supporting this article can be made available upon request from the corresponding author.
