# Supplementary material for: Distinct Neurodevelopmental Trajectories in Groups of Very Preterm Children Screening Positively for Autism Spectrum Conditions
Source: J Autism Dev Disord. 2022 Oct 23;54(1):256–69. doi: 10.1007/s10803-022-05789-4 (PMC10791910; doi:10.1007/s10803-022-05789-4)
Supplement: Supplementary file 1 — Supplementary file1 (DOCX 225 KB) [file 10803_2022_5789_MOESM1_ESM.docx]

**Supplementary Information**

Distinct neurodevelopmental trajectories in groups of very preterm children screening positively for autism spectrum conditions

*eFig. SM1* **Scree plot showing the percentage of variance explained by each principal component**

**
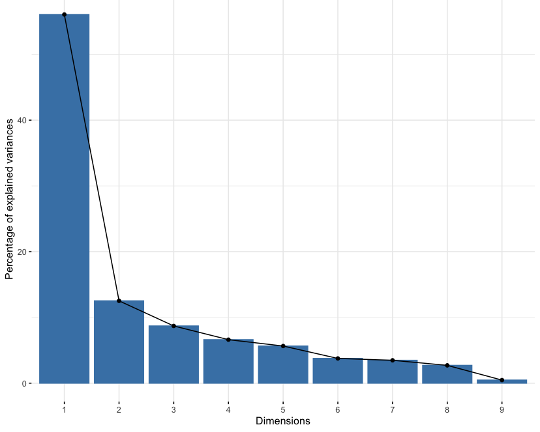
**

*eFig. SM2* **Principal component loadings on Bayley-III and PARCA-R subscale scores**

**
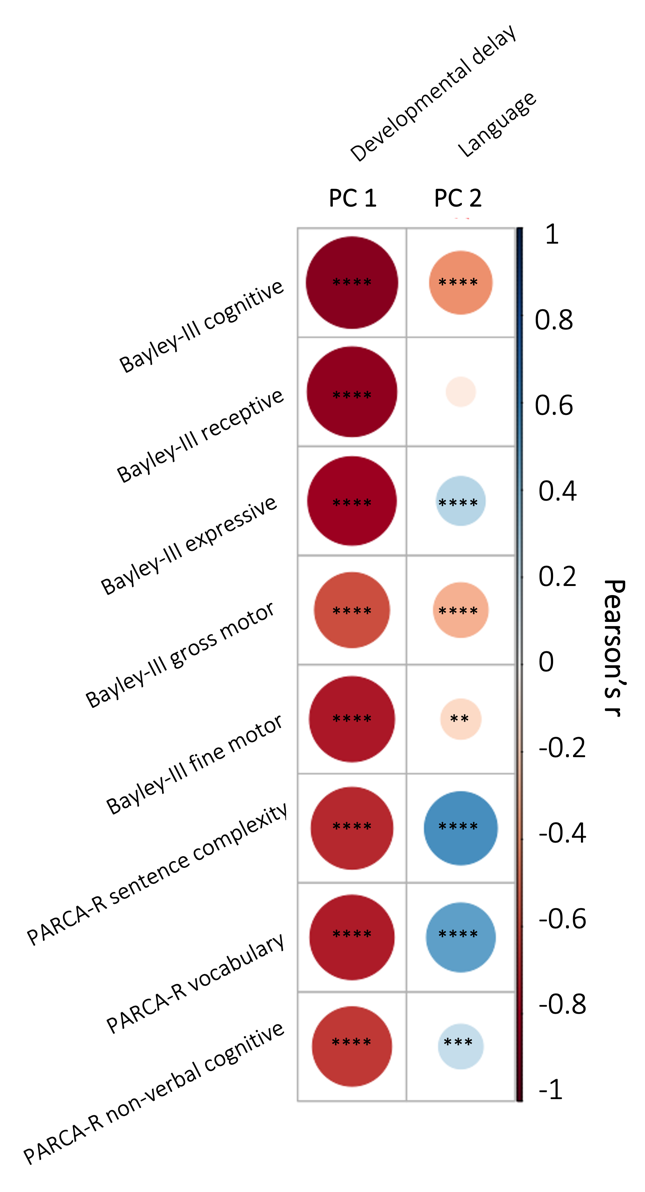
**

eFigure SM2 shows Pearson correlations between each of the two principal components (PC1 and PC2) with Bayley-III cognitive, receptive, expressive, gross motor and fine motor scores and PARCA-R vocabulary, sentence complexity and non-verbal cognitive scores. Positive correlations are indicated in blue and negative correlations in red. Higher correlation coefficients are visualized by darker shades of red/blue and larger circles. **** = p < .0001; *** = p < .001; ** = p < .01; * = p < .05.

**Table SM1**

*ASC traits at 4-7 years and developmental delay at 2-years old in EPT vs VPT born children*

| Variable | Median (Interquartile range) | | F-statistic; p-value |
| --- | --- | --- | --- |
|  | EPT  (N = 132) | VPT  (N = 45) |  |
| SRS-2 SCI | 48.00 (15.00) | 46.00 (10.00) | F = 1.92; p = .166 |
| SRS-2 RRB | 50.00 (16.00) | 48.00 (10.5) | F = 1.32; p = .252 |
| Developmental delay | -0.69 (4.05) | -0.23 (2.78) | F < .001; p = .986 |

Note.

Abbreviations: EPT = extremely preterm. RRB = Restricted Interests and Repetitive Behaviours. SCI = Social Communication/Interaction. SRS-2: Social Responsiveness Scale, Second Edition. VPT = very preterm.

***Post-hoc analyses investigating associations between neonatal brain volumes and ASC traits in childhood***

In order to investigate whether the identified anatomical findings aligned with later high risk for autism, we investigated associations between values reflecting neonatal brain volume (mean Jacobian values extracted from the cerebellum cluster showing a significant difference in the critical group compared to both non-critical and negative subgroups) and later ASC traits (SCI and RRB at 4-7 years; Table SM2). We also conducted the same analyses in the three M-CHAT subgroups (i.e., negative, non-critical positive and critical positive scorers) separately. We found no significant associations between regional cerebellar volume and ASC traits in the entire cohort or in any of the groups (Table SM2).

**Table SM2**

Spearman R coefficients and p-values for correlations between mean neonatal cerebellar log-Jacobian values and childhood SCI/RRB scores

| M-CHAT group | Correlation with SCI  (Spearman R; p-value) | Correlation with RRB  (Spearman R; p-value) |
| --- | --- | --- |
| All participants | R = - .12; p = .115 | R = -.14; p = .070 |
| Negative scorers | R = - .06; p = .539 | R = -.12; p = .175 |
| Non-critical positive scorers | R = -.01, p = .973 | R = 0.09; p = .630 |
| Critical positive scorers | R = -.21; p = .449 | R = -.20; p = .483 |
